# Transactions of the Medical Society of the State of Pennsylvania at Its Thirty-third Annual Session

**Published:** 1883-04

**Authors:** 


**Transactions of the Medical Society of the State of Pennsylvania at its Thirty-third Annual Session,** held at Titusville, May 10th, 11th and 12th, 1882. Volume xiv. Published by the Society. Philadelphia: *Times* Printing House. 1882.

The typographical execution of this volume is creditable to the society, and there is an amount of valuable professional work recorded within its pages which should not lie buried from the sight and observation of the profession. The papers and addresses are able contributions, making the volume a highly creditable addition to the archives of the society and also to the library of its members.

